# Analysis of schizophrenia and hepatocellular carcinoma genetic network with corresponding modularity and pathways: novel insights to the immune system

**DOI:** 10.1186/1471-2164-14-S5-S10

**Published:** 2013-10-16

**Authors:** Kuo-Chuan Huang, Ko-Chun Yang, Han Lin, Theresa Tsao Tsun-Hui, Wen-Kuei Lee, Sheng-An Lee, Cheng-Yan Kao

**Affiliations:** 1 Department of Computer Science and Information Engineering, National Taiwan University, Taipei, Taiwan; 2 Department of Psychiatry, Beitou Branch, Tri-Service General Hospital, Taipei, Taiwan; 3 Graduate Institute of Biomedical Electronics and Bioinformatics, National Taiwan University, Taipei, Taiwan; 4 Department of Information Management, Kainan University, Taoyuan, Taiwan

## Abstract

**Background:**

Schizophrenic patients show lower incidences of cancer, implicating schizophrenia may be a protective factor against cancer. To study the genetic correlation between the two diseases, a specific PPI network was constructed with candidate genes of both schizophrenia and hepatocellular carcinoma. The network, designated schizophrenia-hepatocellular carcinoma network (SHCN), was analysed and cliques were identified as potential functional modules or complexes. The findings were compared with information from pathway databases such as KEGG, Reactome, PID and ConsensusPathDB.

**Results:**

The functions of mediator genes from SHCN show immune system and cell cycle regulation have important roles in the eitology mechanism of schizophrenia. For example, the over-expressing schizophrenia candidate genes, SIRPB1, SYK and LCK, are responsible for signal transduction in cytokine production; immune responses involving IL-2 and TREM-1/DAP12 pathways are relevant for the etiology mechanism of schizophrenia. Novel treatments were proposed by searching the target genes of FDA approved drugs with genes in potential protein complexes and pathways. It was found that Vitamin A, retinoid acid and a few other immune response agents modulated by RARA and LCK genes may be potential treatments for both schizophrenia and hepatocellular carcinoma.

**Conclusions:**

This is the first study showing specific mediator genes in the SHCN which may suppress tumors. We also show that the schizophrenic protein interactions and modulation with cancer implicates the importance of immune system for etiology of schizophrenia.

## Background

Recent studies suggest that schizophrenia may result from neuropathological abnormalities and imbalanced immune systems. Signal transduction dysfunction of the neuroendocrine system are responsible for schizophrenia, especially the dopamine, serotonin and glutamate system in the temporal and frontal lobe of the brain area [1,2]. Although an increasing number of studies show that the immune-mediated mechanism for inflammation responses are the pathogenesis of schizophrenia [3], the corresponding specific complexes, pathways and candidate genes are not well-documented for the etiological model of schizophrenia.

In recent years, there have been many studies focusing on the discovery of schizophrenic candidate genes and the construction of PPI networks and related pathways for the hope of a better understanding of schizophrenia. However, genetic association researches have been published with largely inconsistent results [4]. It was generally believed that a protein sub-network, rather than a single gene or genetic variants, accounts for the susceptibility of schizophrenia. Sun J. et al. (2008) surveyed the increased association studies from the SchizophreniaGene database in ethnic populations [5], in which candidate genes are selected and ranked by the combined odds ratio method as an important index of the candidate genes [6]. It provides a basis for the investigation of molecular and cellular mechanisms of schizophrenia by the analysis of gene features for a genetic network. A regularly updated online database of genetic association studies for schizophrenia (SZGene) was collected from Allen NC. et al. (2008)[4]. Sun J. et al. (2010) [7] selected a list of schizophrenia candidate genes by a multi-dimensional evidence-based approach to provide a comprehensive review of the schizophrenia molecular networks. The identified pathway characteristics of schizophrenic candidate genes have important implications of molecular features for schizophrenia. Another gene risk prediction study used the translational convergent functional genomics approach introduced by Ayalew M. et al. (2012) to prioritize schizophrenia genes by gene-level integration of genome-wide association study data to identify top candidate genes [8]. These candidate gene studies conclude the specific genetic variants or patterns contributing to the schizophrenic model by integrating functional and genotypic data. The previous literatures provide different databases and integration of formulated reliability analysis, ranking and scoring for important candidate genes of schizophrenia.

Schizophrenic patients have less chance to develop cancer than the general population [9]. Lower incidence of cancers, especially in lung, prostate and bladder cancer, was found in schizophrenic patients [10-12]. Research suggests that cancer risk decreases as the duration and age of onset of schizophrenia increases [13]. Cancer protective factors in schizophrenic patients are genetic predisposition [14,15]. These literature reviews have implication of sharing common disease genes or pathways between schizophrenia and cancer, and that schizophrenia is a protective factor for cancer [16].

To demonstrate the genetic relationship between schizophrenia and cancer, network biology and systemic bioinformatics data such as protein-protein interactions (PPIs) and related pathways were introduced. The data of human PPIs brought insights to the network biology of diseases and explained the interrelationships among disease-related genes and proteins. Through the development of modulation interaction networks of schizophrenic candidate genes, the related resources of molecular biology were integrated to explore the molecular biological information of disease mechanism and related drug targets or complexes.

Efforts on the exploration of schizophrenic common pathways from corresponding candidate gene analysis are gaining more attention and represent for novel treatment approaches in schizophrenia. Postulated disease networks are analyzed by tools or algorithms such as modularity, centrality (closeness and degree) and clique analysis derived from network biology, which the functional relevance of different gene sets and related biological significance were analyzed. In functional genomics, there are available integrative protein interaction databases developed to identify gene sets of interest which involve similar disorders. These gene sets are commonly presented as gene modules, protein complexes or pathways such as in the Database for Annotation, Visualization and Integrated Discovery (DAVID) [17], Kyoto Encyclopedia of Genes and Genomes (KEGG) [18] and ConsensusPathDB [19]. In these integrative databases, candidate gene sets from disease-related network to gene ontology classification were mapped to the related molecular pathways and PPI networks.

This study integrates comparative analysis of different genetic research results. From the RNA extraction of microarray data, the expression level of each gene was acquired from BA22-derived brain cells and hepatocellular carcinoma cells. Generated from two group sets of candidate genes, the corresponding PPI networks were constituted and analyzed. The over- and under- expression level of genetic interactions between schizophrenia and hepatocellular carcinoma are not only found by the direct effect of inhibition of candidate genes for cancer, but also through an indirect modulation of protein-protein interactions in the cancer genetic network which have potential effects on tumor suppression by analysis of the core schizophrenia-cancer genetic network. The differences in gene expression and PPI sub-networks between schizophrenia and hepatocellular carcinoma were analyzed to discover protein complexes and possible drug targets.

## Methods

**Figure 1:**
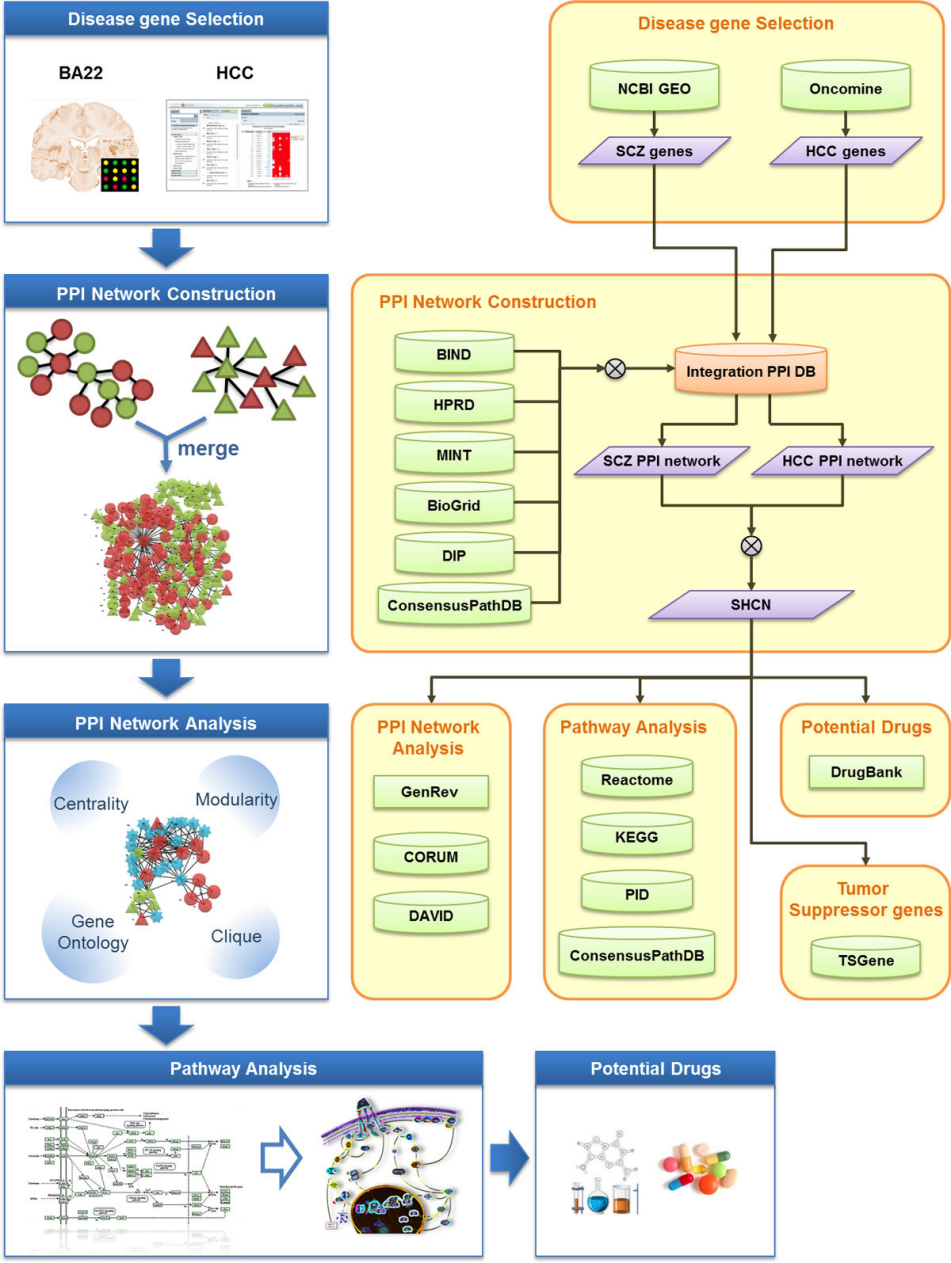
**Flow chart of research protocol**. By analysis of BA22 microarray data and Roessler liver 2 sample, the significant candidate genes were screened and constructed the PPI network for schizophrenia and cancer. The potential complexes or drugs are discovered by their corresponding pathways. The right part shows the corresponding tools used for analysis of candidate gene resources, PPI networks and pathway databases, the potential pathways and drugs.

By analyzing microarray data of Brodman Area 22 (BA22), susceptible genes for schizophrenia were proposed. Through the analysis of the PPI network of schizophrenia and cancer, the potential complexes or possible drugs were proposed. The research flow is shown in Figure 1. The related tools for candidate gene resources, PPI networks and pathway databases with analytic tools or algorithms are described in the following sections.

### Schizophrenia related genetic information

Schizophrenic candidate genes from literature studies such as Sun J. et al. and Ayalew M. et al. were selected based on the chromosome classification, mapping genetic literature data and statistic measures, and the highly relevant genes were sorted with different ranking systems [7,8]. Ayalew M. et al. analyzed the schizophrenic candidate genes by ranking and scoring the relevant candidate genes from NCBI literature [8].

### Selection of schizophrenic candidate genes by microarray data

Human BA22 of the prefrontal cortex is believed to be responsible for many positive symptoms and cognitive dysfunction in patients with psychiatric illness. RNA was extracted from post-mortem BA22 tissue from schizophrenic and control patients. The RNA samples were analyzed by Affymetrix GeneChip HG-U133 Plus2.0. We downloaded the microarray data (GSE21935) from the NCBI GEO database [20]. This dataset consists of 19 control and 23 schizophrenia samples.

The over- and under-expression genes in the BA22 samples were selected using the Student's t-test between the schizophrenia and control samples. The genes of the corresponding probes with p-value < 0.05 were defined as abnormally expressed and proposed as the candidate genes for schizophrenia.

### Cancer-related genes by microarray

The expression data for human cancer including breast cancer, leukemia, colon cancer and prostate cancer is collected in the ONCOMINE database (http://www.oncomine.org/). This database currently contains 674 datasets and information of 73327 tissue samples (ONCOMINE version 4.4.3).

The Roessler liver 2 sample [21] includes 445 samples which contains 225 hepatocellular carcinoma and 220 liver samples, a total of 12624 mRNA expressions are measured by the Human Genome U133A 2.0 Array and the data is released on 2009/11/1 by ONCOMINE [22]. 126 over- and 126 under-expression genes are selected which respectively account for 1% of top candidate genes from the Roessler liver 2 sample of ONCOMINE. The over- and under- expression candidate genes from BA22 samples and the Roessler liver 2 samples are listed in Additional file 1.

### Construction of schizophrenia and cancer network

In order to construct a PPI network, the fundamental basis of human PPI network was formulated by the integration of interaction databases including BIND [23], HPRD [24], MINT [25], BioGrid [26], DIP [27] and ConsensusPathDB [19]. ConsensusPathDB currently contains the most comprehensive publicly available repository including genes, proteins and complexes interaction for Homo sapiens.

In PPI networks, each node represents an encoded gene and each edge represents a protein interaction by literature reviews or experiments. The interaction network with significant functionalities generates genetic network through the selection of different query genes, such as Level-One PPI (L1PPI) and Query-Query PPI (QQPPI). QQPPI networks include only the query marker genes as the nodes and show direct interactions among these queries. L1PPI networks also show other non-query nodes directly connected to the queries. L1PPI network allowed analysis of an extended network and indicated indirect interactions [28].

**Figure 2:**
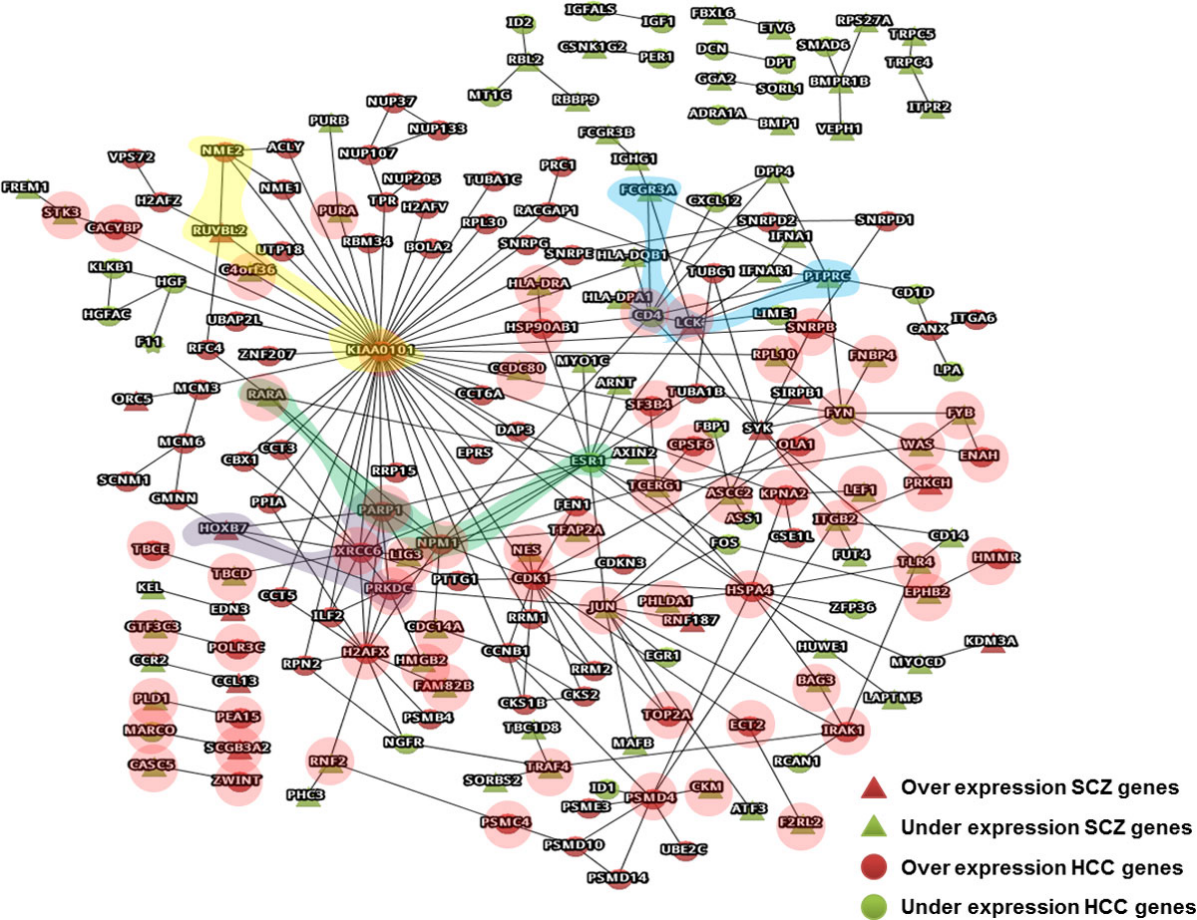
**The over- and under-expression PPI network for schizophrenia and hepatocellular carcinoma**. Red nodes denote over-expression genes; green nodes represent under-expression genes, circle nodes indicate hepatocellular carcinoma genes, triangular nodes indicate schizophrenia genes. The under-expression gene sets between schizophrenia and hepatocellular carcinoma genes are noted including CD4-CXCL12-DPP4, HLA-DPA1-CD4-FCGR3-PTPRC, MYO1C-ESR1-RARA, FOS-JUN-EGR1, etc.

The over- and under-expression candidate genes from schizophrenia and hepatocellular carcinoma were combined into a single gene list, defined as SHCGene. The SHCN is defined as the L1PPI and QQPPI networks using the SHCGene as the query genes. The QQPPI of SHCN in Figure 2 illustrates the direct relationship of protein-protein interaction between the two diseases. In order to find the mediator genes between diseases, the L1PPI of SHCN was analyzed to find the mediators of query genes. By the comparison of different mediators, the potential core modulation network for schizophrenia was effectively extracted.

### Selection of candidate genes, complexes and modularity from SHCN

GenRev [29] was used to construct the functional modularity of schizophrenia by calculating the betweeness and closeness centrality of genes. The gene included by the QQPPI of SHCN were used as the input for GenRev to construct a reference network and define the importance of genes using sub-network analysis by calculating MCL [30]. Combined with the SHCGene, the candidate genes collectively represented highly modulating functionality or inhibitory relationship between the diseases [31]. Using the clique analysis network algorithm, the involving complexes were extracted from the PPI network.

### Analysis of schizophrenic pathways and drugs

By analysis and verification of known pathways, image information resource (http://www.sabiosciences.com/pathwaycentral.php) provided by pathway central presents the pathway information related to schizophrenia. The current available pathway databases for analyzing modulation network mechanisms include KEGG [18], Reactome [32], PID [33] and ConsensuspathDB[19]. ConsensuspathDB enables analysis of different types of functional interactions between genes and regulatory pathways [34] through integration of meta-databases such as KEGG, Reactome and PID.

The interactors of SHCN were mapped to the respective pathways of ConsensusPathDB which provide corresponding information from original pathway databases. The corresponding detailed pathway information from each pathway database were searched for the relationship between pathways and SHCN. The significant pathways from the pathway enrichment analysis test involving genes from the L1PPI of SHCN. The corresponding genes from the pathway databases were then prioritized. The importance of the respective pathways was evaluated by p-values [35].

By integration of schizophrenic related genes and pathways, novel drugs were discovered for further investigation. The target genes for specific drugs or complexes for query gene functions and interactions were searched against DrugBank [36] which lists FDA-approved agents and target complexes.

### Potential drugs or complexes related to tumor suppression

In order to analyze the potential complexes or targets which might have tumor suppression effects, the clique complexes were searched against CORUM [37] to find significant protein complexes. Tumor suppression genes were collected for comparison from TSGene [38].

## Results

### Schizophrenia candidate genes related to tumor suppression

Despite the increased risk factors for the schizophrenic patients such as heavy smoking, poor diet habit and inadequate physical activities, the protective factor for cancer incidence in schizophrenic patients [16] such as TP53 and APC, which plays a key role in the susceptibility of schizophrenia and the reduced cancer risk by apoptosis [39-42], have implication of the explanation for less incidence of cancer for schizophrenic patients. Therefore, to see the differential interaction between diseases in the PPI network, schizophrenic candidate genes were examined from three different literature results and compared with the BA22 over- and under-expression genes with tumor suppression. Of the 716 tumor suppression genes, 26 genes were contained in Sun J. et al.(2008), 38 genes were contained in Allen NC. et al. (2008) and 47 genes were contained in Ayalew M. et al.(2012), this implicates that schizophrenia candidate genes are related to the tumor suppression genes and share a common genetic biological regulation. A total of 7.6% tumor suppression genes also appeared in schizophrenia candidate gene list. ZFHX3, RND3, KLF5, ERBB4, EGR1 and APC genes are both schizophrenia candidate genes and tumor suppression genes (Additional file 2). From the genetic aspect, the genes which were contained in both the schizophrenia candidate gene list and tumor suppression gene list have implication of protective genetic interaction relationship between schizophrenia and cancer.

### The overlapped genes in schizophrenic genetic network

The schizophrenic candidate genes reported by the literature reviews of Sun J. et al.(2008), Allen NC. et al.(2008), Ayalew M. et al.(2012) and BA22 microarray samples are inconsistent, which might be attributed to the ethnic difference in allele and haplotype frequencies [43]. Furthermore, when comparing the 781 genes of the SZGene database from Allen NC. et al. (2008) [4], the 183 candidate genes from Ayalew M. et al.(2012)[8] and the 75 candidate genes from Sun J. et al.(2008) [5], only 1 overlapped gene (BDNF) was found, and only 36 overlapped genes appeared in any two of three sets. However, when we extended the previous three gene lists using L1PPI analysis, we obtained 10679, 4109, and 1821 PPIs and 4386, 2611, and 1438 interactors for the 781, 183, and 75 genes, respectively. As a result, there are now 663 interactors co-existing in the three L1PPI extended gene lists, and 1520 interactors co-existing in any two of three L1PPI extended gene lists. The overlapping candidate genes in the three original sets range from 0.12% to 1.4%; however, the overlapped interactors range from 15% to 46%, which indicate the importance of mediator genes (Additional file 3) in the candidate genetic network of schizophrenia rather than query genes as key roles of disease susceptibility.

### Analysis of the schizophrenic genetic network with different expression levels by human protein-protein interactions

By analyzing the microarrays of human BA22 samples, the over- and under-expression network for schizophrenia and hepatocellular carcinoma reflect the interaction of both diseases by annotation of each different node with over- and under-expression features. A total of 472 genes including 138 over-expression genes and 334 under-expression genes are selected from the BA22 samples with a p-value less than 0.05, derived 3247 interacts of direct interactors of L1PPI for schizophrenia.

Direct interaction genes observed from QQPPI interaction formulates potential common functional modularity between schizophrenia and cancer. The genes which are contained in both the schizophrenic candidate gene list and the hepatocellular carcinoma candidate gene list from the Roessler liver 2 sample constitutes 197 over- and under-expression level genes and 264 PPIs in the QQPPI of SHCN network **(**Figure 2**)**.

Many under-expression gene sets between schizophrenia and hepatocellular carcinoma genes are noted, including CD4-CXCL12-DPP4, HLA-DPA1-CD4-FCGR3-PTPRC, MYO1C-ESR1-RARA, FOS-JUN-EGR1, etc. Moreover, the combination of over- and under-expression level genes of both diseases such as CASC5-ZWINT, MARCO-SCGB3A2, PLD1-PEA15, GTF3C3-POLR3C, TBCE-TBCD-XRCC6, etc.(pink region) highlight the potential biological significance of gene set combination implicating the protective factor for both diseases. In the schizophrenia-hepatocellular carcinoma network (SHCN), the numbers of under-expression genes exceed the over-expression genes for schizophrenia. Furthermore, the under-expression schizophrenia genes interact with the over-expression genes of hepatocellular carcinoma (Figure 2) which have the implication of a genetic modulation mechanism for both diseases. These gene sets might have important roles in potential cellular modulation or neurodevelopmental regulation functions of disease pathophysiological mechanism by their involvement in the molecular pathways of related complexes.

### Modularity and complex analysis of SHCN

Direct interaction genes could be easily observed from the QQPPI of SHCN and proposed as significant common functional modularity from the potential modulation process of both diseases which may have a lot of hidden biological significance. GenRev analysis showed that the significant genes and functional modularity were discovered from the QQPPI of SHCN by the expression level of each gene input as the score of GenRev. SYK and LCK genes were calculated with highest betweeness and ranked as top genes for the SHCN. The discovery of functional modularity by the MCL algorithm is listed in Table 1. With the use of systemic and integrated analysis of functional annotation information by using the DAVID, novel enriched gene list with functional modularity was analyzed. The functional modularity from GenRev can be mapped to DAVID to annotate the potential functions involving T cell and lymphocyte activation, cell mediated cytotoxicity, DNA and calcium binding and inflammatory response. The over-expression schizophrenia genes involving the SYK and LCK genes play a key role in functional modularity in T cell, leukemia and lymphocyte activation which is crucial in immune-related responses.

**Table 1:** Modularity analysis from SHCN

Gene module by MCL algorithm	Functional annotations by DAVID
DPP4, FCGR3A, PTPRC, IFNAR1, IFNA1, LCK	T cell activation, leukocyte and lymphocyte activation, cell surface receptor linked signal transduction Natural killer cell mediated cytotoxicity
ATF3, RNF187, MAFB, JUN, ASCC2	Transcription factor activity, DNA binding, regulation of transcription
FUT4, ITGB2, TLR4, CD14, PRKCH	Inflammatory response, receptor complex
VEPH1, BMPR1B, RPS27A	Cell part
TCERG1, WAS, FYB	Unknown
TRPC4, TRPC5, ITPR2	Calcium binding, intrinsic to membrane
TBC1D8, TRAF4, SORBS2	Alternative splicing

Common mediator genes of both diseases could be easily observed from the L1PPI of SHCN. Clique analysis formulates common functional modules and enables easy screening of the co-expression or functional units of gene sets which maps to related target complexes or common pathways of biological function [28,31]. As shown in Figure 2, three clique-4 gene sets were derived from SHCN: CD4-FCGR3A-LCK-PTPRC (sky blue region), ESR1-NMP1-PARP1-RARA (green region) and HOXB7-PARP1-PRKDC-XRCC6 (purple region). The corresponding complexes were retrieved from CORUM[37] and are shown in Table 2, which mediate genetic functions such as cell cycle, transcriptional activation and immune responses. These complexes involve potential modulating mechanism for the discovery of novel treatment agents.

**Table 2:** Clique-4 complexes in SHCN

Complex name	Functional annotation by DAVID
CBP-RARA-RXRA-DNA complex	RNA synthesis, transcriptional control and activation
CD20-LCK-FYN-p75/80 complex	Cellular signaling transduction, protein tyrosine kinase
LCK-SLP76-PLC-gamma-1-LAT complex	Transmembrane receptor protein tyrosine kinase signaling pathways, immune response
MDC1-H2AFX-TP53BP1 complex	Mitotic cell cycle and cell cycle control, DNA damage response
NCOA6-DNA-PK-Ku-PARP1 complex	Cell cycle and DNA processing, DNA recombination and DNA repair
Notch1-p56LCK-PI3K complex	RNA synthesis, transcription activation and control
P53-BARD1-Ku70 complex	Cell death, apoptosis
RC complex	Cell cycle, DNA synthesis and replication

**Figure 3:**
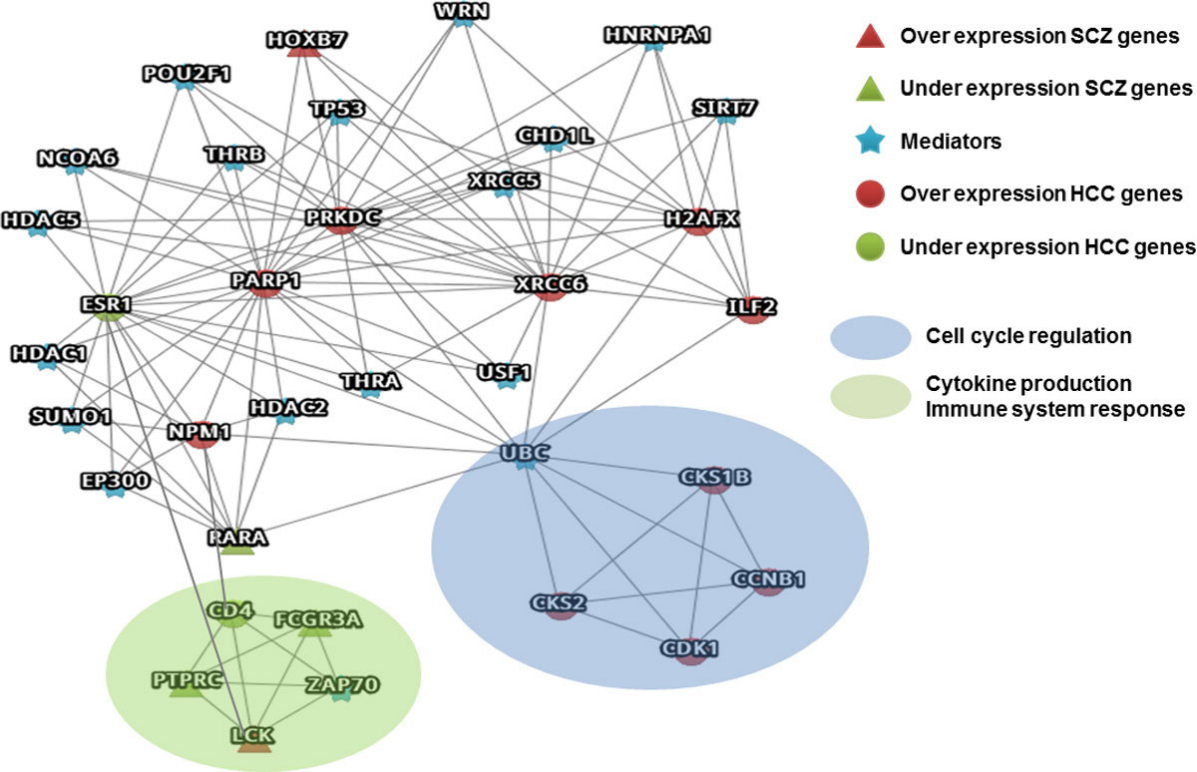
**Clique-5s for potential complexes in SHCN**. Blue star nodes indicate mediators; red nodes represent up-expression genes; green nodes represent under-expression genes; circle nodes indicate hepatocellular carcinoma genes; triangular nodes indicate schizophrenia genes: (a) The CD4-FCGR3A-ZAP70-LCK-PTPRC clique formulates the functional module of cytokine production related to the immune system response. (b) The CKS1B-CCNB1-CDK1-CKS2-UBC clique is responsible for cell cycle regulation.

Furthermore, the L1PPI of SHCN formulates the critical functional modules and mediators by clique analyses. The most extended sub-network is clique-5. Figure 3 presents the 34 genes with 118 PPIs in the clique-5 sub-network. Two gene groups are identified. One is the clique CD4-FCGR3A-ZAP70-LCK-PTPRC which formulates the functional module of cytokine production related to immune system response; the other is the clique CKS1B-CCNB1-CDK1-CKS2-UBC which is responsible for cell cycle regulation. The schizophrenia-related SNP study supports the significant result of the involving pathway of translocation of ZAP-70 to immunological synapse by Reactome [44].

UBC and TP53 are mediator genes which are potential targets involved in the disease mechanism for schizophrenia and cancer[45-48] which appears in the clique-5 network. PRKDC, PARP1, NPM1 and XRCC6 are hepatocellular carcinoma over-expression genes which modulate cell cycle regulation network through the modulation of UBC[49]. HOXB7 and RARA are schizophrenic genes with different gene expressions, modulated through the retinoid signaling pathway by the hepatocellular carcinoma over-expression gene PARP1 [50]. Furthermore, these genes formulate an important genetic functionality of the immune system, which illustrate the relationship between schizophrenia and autoimmune diseases.

### The immune-related pathway responsible for pathological mechanism of schizophrenia

In order to prioritize the potential pathways in which schizophrenia and hepatocellular carcinoma candidate genes are involved, the L1PPI extended over- and under-expression genes from BA22 sample and Roessler liver 2 sample were used to search for significant pathways in PID. The crucial pathways are listed in Additional file 4 which is ranked by the p-value and FDR-adjusted p-value by the Benjamini-Hochberg procedure. The top ranked pathways with significant p-value associated with BA22 and Roessler liver 2 sample include the PDGFR-beta signaling pathway, the ErbB1 downstream signaling and BCR signaling pathway which indicates common pathways for schizophrenia and cancer. It is appealing that another group of significant pathways including the Fc-epsilon receptor I signaling in mast cells, the TCR signaling in CD4+ T cells and IL2-mediated signaling events highlight the importance of immune system mediated pathways in the key role of schizophrenia susceptibility.

**Figure 4:**
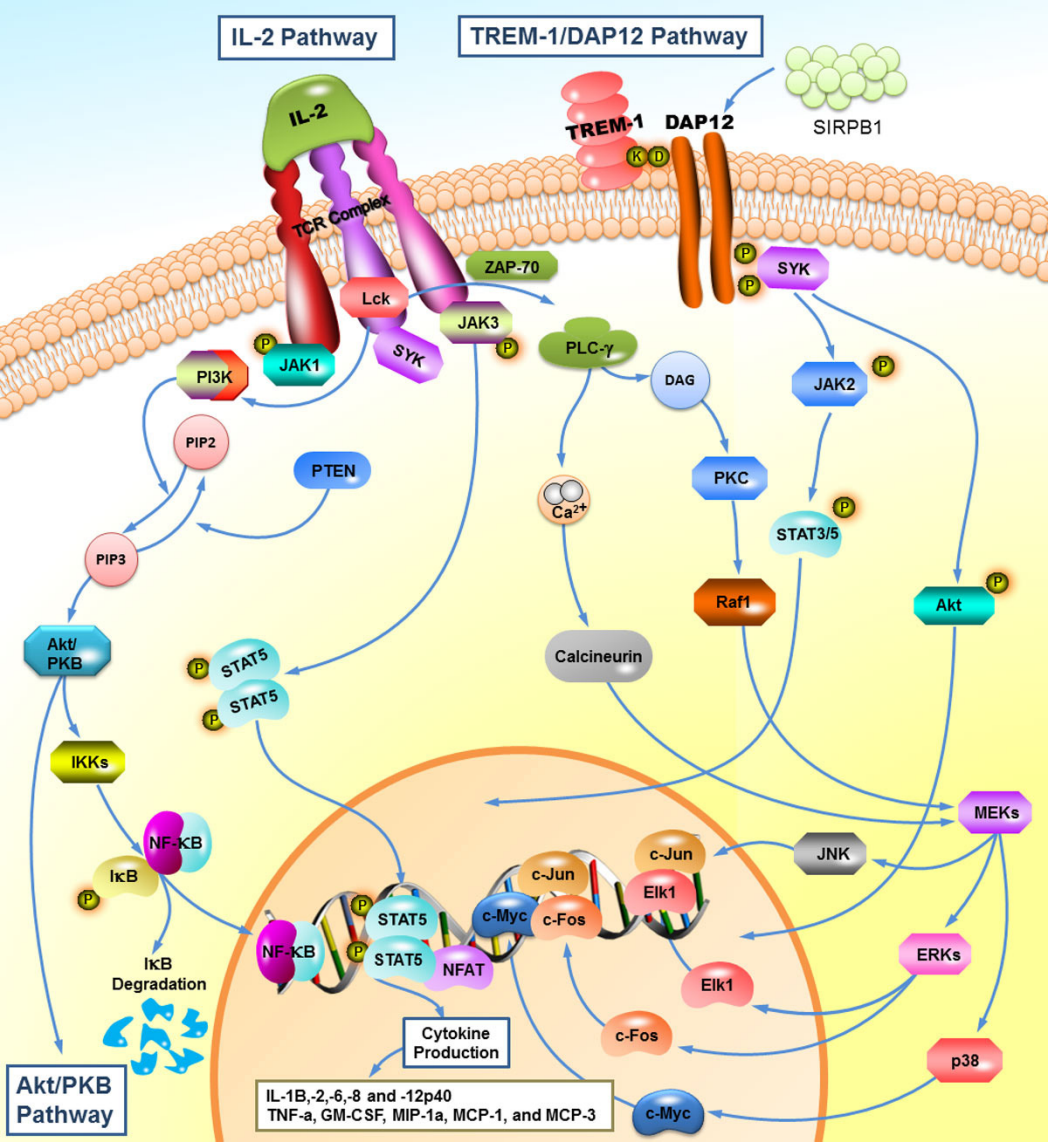
**IL-2 and TREM-1/DAP12 pathway**. A specific gene set of schizophrenic over-expression genes includes SIRPB1, LCK and SYK. Strong correlations with immune system pathways were found which involve the IL-2 and TREM-1/DAP12 pathway which are responsible for etiology mechanism for schizophrenia in PID. The SIRPB1 protein interacts with the glycoprotein DAP12 to induce the signal transduction and the formation of the SIRPB1/DAP12 complex, and then transduces signals to the nucleus with the activation of SYK. Activation of DAP12 and triggering of the TREM-1 receptor result in the production of pro-inflammatory cytokines including interleukin family cytokines, TNF-a, and GM-CSF, etc.

A specific gene set of schizophrenic over-expression genes includes SIRPB1, LCK and SYK (Figure 2). SYK and LCK were the over-expression genes with high betweenness, however, SIRPB1 was an over-expressed gene for schizophrenia and directly linked to SYK from the QQPPI of SHCN, and it may have an implication of a potential disease mechanism for schizophrenia. Another responsible pathway involves the IL-2 pathway which Interleukin-2 binds to the IL-2 receptor to activate LCK and SYK, which helps explain that the over-expression genes: SIRPB1, LCK and SYK might be responsible for one of the possible disease mechanisms for schizophrenia. The corresponding pathways containing SIRPB1, LCK and SYK were retrieved from KEGG, PID and Reactome. Strong correlations with immune system pathways were found which involve the IL-2 and TREM-1/DAP12 pathway which are responsible for the etiology mechanism for schizophrenia in PID (Figure 4). The SIRPB1 protein interacts with the glycoprotein DAP12 to induce signal transduction and formation of the SIRPB1/DAP12 complex, and then transduces signals to the nucleus with the activation of SYK. Activation of DAP12 and triggering of the TREM-1receptor result in the production of pro-inflammatory cytokines and receptor expression on natural killer (NK) cells, monocytes and neutrophils with degranulation of neutrophilic granules and phagocytosis.

Another responsible pathway involves the IL-2 pathway in which Interleukin-2 binds to the IL-2 receptor to activate LCK and SYK, then induces cascade signal transduction through Rho, PI3K and Akt/PKB signaling pathways. The results help to explain that the over-expression genes SIRPB1, LCK and SYK are responsible for one of the possible disease mechanisms for schizophrenia. The immune-related pathways reveal the novel discovery for the treatment and pathophysiological mechanism for schizophrenia.

### Discovery of candidate drugs or treatments for both schizophrenia and cancer

**Figure 5:**
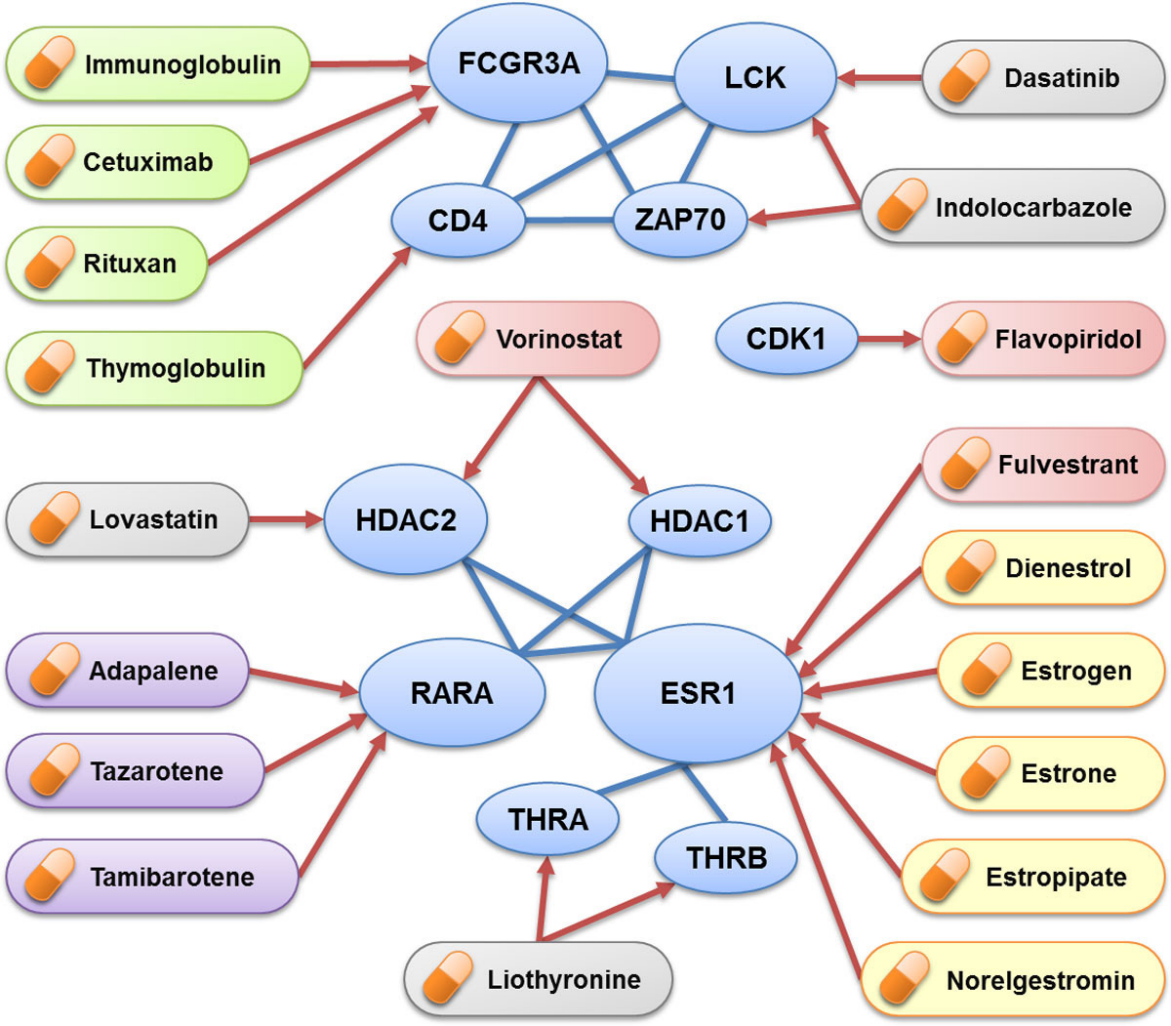
**Drugs and related genes**. Potential candidate drugs are mapped to the L1PPI of SHCN using clique analysis and prioritized using DrugBank data. (A) The drugs with green background are immune-related drugs. (B) The drugs with red background are anti-cancer drugs. (C) The drugs with yellow background are sex-hormone drugs. (D) The drugs with purple background are retinoid drugs.

The FDA-approved potential candidate drugs mapped from L1PPI of SHCN by the clique analysis and prioritized from DrugBank are listed as Table 3. The relationship between candidate drugs and encoded genes are illustrated in Figure 5. Cetuximab, Immunoglobulin, Rituxan and Thymoglobulin are immune-related drugs encoded by FCGR3A and CD4 genes in which Cetuximab is an epidermal growth factor receptor (EGFR) inhibitor used for the treatment of metastatic colorectal cancer and head and neck cancer [51,52]. The use of Rituxan(Rituximab) has been proposed for patients with B-cell non-Hodgkin's lymphoma (NHL) and B-cell chronic lymphocytic leukaemia (CLL) [53,54]. It shows that the immune-related drugs with the potential of antibody therapy are associated with several mechanisms of potential treatment for cancer including interference of vital signaling pathways by tumor-bound antibody through the Fc portion of the antibody [55].

**Table 3:** Potential drugs discovered from Clique-5 network

Drug ID	Gene Name	Drug Name	Drug Class
DB00002	FCGR3A	Cetuximab	Immune
DB00028	FCGR3A	Immunoglobulin	Immune
DB00073	FCGR3A	Rituxan	Immune
DB00098	CD4	Thymoglobulin	Immune
DB00210	RARA	Adapalene	Retinoids
DB00227	HDAC2	Lovastatin	Cholesterol-lowering agent
DB00269	ESR1	Estrogen	Sex hormone
DB00279	THRA	T3, liothyronine	Thyroid hormone
DB00279	THRB	T3, liothyronine	Thyroid hormone
DB00655	ESR1	Estrone	Sex hormone
DB00799	RARA	Tazarotene	Retinoids
DB00890	ESR1	Dienestrol	Sex hormone
DB00947	ESR1	Fulvestrant	Anti-cancer
DB01254	LCK	Dasatinib	Tyrosine kinase inhibitor
DB02010	LCK	Indolocarbazole	Protein kinase C inhibitor
DB02010	ZAP70	Indolocarbazole	Protein kinase C inhibitor
DB02546	HDAC1	Vorinostat	Anti-cancer
DB02546	HDAC2	Vorinostat	Anti-cancer
DB03496	CDK1	Flavopiridol	Anti-cancer
DB04574	ESR1	Estropipate	Sex hormone
DB04942	RARA	Tamibarotene	Retinoids
DB06713	ESR1	Norelgestromin	Sex hormone

The sex hormones, estrogen and thyroid hormone (T3), are candidate drugs involving THRA, THRB and ESR1 in the SHCN. Dasatinib and Indolocarbazole are protein kinase inhibitors involving the LCK and ZAP70 genes. Vorinostat and Flavopiridol are anti-cancer agents involving the HDAC1, HDAC2 and CDK1 genes. Lovastatin involving HDAC2 is a cholesterol-lowering agent. These candidate drugs mostly act as cancer treatment agents but have little evidence for treatment of schizophrenia.

Adapalene, Tazarotene, Tamibarotene are retinoids which involved RARA gene with multiple functions including eye vision, immune function, and activation of tumor suppressor genes. The retinoid acid has been reported for cancer prevention and treatment of leukemia. Promising results by Fenretinide (retinoid acid) in breast cancer prevention provides a strong rationale for cancer treatment especially in combination with chemotherapy in non-small cell lung cancer [56].

The retinoids with the promising role in chemoprevention of premalignant lesions in the head and neck have been the focus of cancer intervention treatment [57] which Stra6 unregulated RA-responsive genes unregulated by DNA damage with important role in cell death responses. Novel findings between the retinoid acid and TP53 pathways provide a new insight which enhances the tumor suppression functions. It implicates the significant role of vitamin A metabolites in cancer prevention and treatment [58]. Moreover, Vitamin A (retinol), the biologically active form of retinoic acid, has been proposed to be involved in the pathogenesis of schizophrenia by the genetic basis of encoding retinoid acid metabolism enzymes. Seven genes were investigated and involved in the synthesis, degradation and transportation of RA, ALDH1A1, ALDH1A2, ALDH1A3, CYP26A1, CYP26B1, CYP26C1 and Transthyretin (TTR), for their roles in the development of schizophrenia [59]. The expression of the transthyretin (TTR) tetramer, which is a retinoid transporter, is increased significantly in the plasma of schizophrenic patients. Retinoid dysfunction might be involved in the pathology of schizophrenia[60].

## Discussion

Few microarray studies of mental disorders have used post-mortem brain samples of human species from schizophrenic patients. Researchers did not have convenient access to brain samples of psychiatric patients until 1994 when the Stanley Brain Collection started. However, the result of using a single expression dataset could be biased. A more diverse microarray dataset of BA22, BA10, BA46 samples and other tissue specific samples from schizophrenic patients could be compared and analyzed for the future study.

In order to determine the extent of which the BA22 genetic network is the result of chance, we introduced 5000 randomly generated control networks by randomly selecting 472 genes from 32560 official human genome gene symbols. The 472 query genes generated from the BA22 sample formulate a genetic network with 15 subgraphs and 36 QQPPIs. However, the mean subgraphs and QQPPIs of 5000 randomly generated networks was 7.54 and 18.54 respectively. The number of QQPPIs of the BA22 genetic network ranked top 4.52% of all randomly generated networks, which shows that the BA22 genetic network unlikely to be the result of chance.

In the search of schizophrenia specific pathways, there are consistent results compared with Sun J. et al. (2010) that there are 4 among 8 pathways involving in or related to the immune system [7] including the glucocorticoid receptor regulatory network, the Fc-epsilon receptor I signaling in mast cells, the NF-kappaB pathway and IL-10 signaling. The interleukin family (IL1, IL2, IL3, IL4, IL5, IL6, IL8, IL10, IL12, IL23 and IL27) pathways also implicate significant schizophrenic pathways. These immune-related pathways with significant p-value (<0.01) supports the autoimmune hypothesis of schizophrenia [61].

### Schizophrenia and the immune system

A higher prevalence of several autoimmune disorders has been reported in schizophrenic patients. A growing evidence of researches suggests that the human immune system is associated with the susceptibility and increased risk for schizophrenia, which alterations in the inflammation process and cytokine production have been focused as important mediators in the inflammatory process. Alteration of the immune system and increased level of cytokine are also associated with schizophrenia [62,63]. However, evidence for common genetic susceptibility between schizophrenia and autoimmune disorders is mostly indirect and not intuitive. On the molecular level, schizophrenia and autoimmune disorders seem to share specific genes with family predisposition [64].

Accumulated evidence has identified abnormalities of the immune system in schizophrenia patients. Neuroinfalmmatory and arachidonic acid cascade markers are increased in schizophrenic patients [65]. Dysregulation of the alternative complement pathway in schizophrenia patients provides evidence that the imbalance of immune system contribute to schizophrenia [66].

### Putative association of SIRPB1-LCK-SYK genes in SHCN

The LCK gene encodes a 56-kDa protein-tyrosine kinase, predominantly expressed in T lymphocytes, crucial for initiating T cell antigen receptor (TCR) signal transduction pathways is associated with phosphorylation of the T cell antigen receptor(TCR) by tyrosine kinase which is an essential step in the activation of T cell [67]. Isothiazolinones is a kind of fungicidal and bactericidal effect with properties of broad spectrum, which can quickly inhibit microbe growth, leading to the death of microbes. It is also a novel inhibitors of p56(LCK), which is identified to inhibited kinase activity [68]. TCR-induced stimulation of T cells led to simultaneous phosphorylation of p56(LCK) residues at Y505 and Y394 [69]. Serial activation of the tyrosine kinases LCK and ZAP-70 initiates signaling downstream of the T-cell receptor. ZAP-70 and SYK which is essential for B-cell receptor signaling, share a unique domain structure for protein kinases and undergo conformational change on binding to doubly phosphorylated ITAM peptide [70].

In summary, the SIRPB1 mediated LCK and SYK gene activation are associated with schizophrenia related to BA22 tissue specific gene. The figure illustrates the use of genetic network analysis as an explanation for potential mechanisms of schizophrenic pathway.

### Schizophrenia and IL-2/TREM-1 pathway

The cellular and molecular module for immune system involving IL-2 pathway and TREM-1/DAP12 pathway were proposed for potential susceptibility for schizophrenia. Recent study proposed strong evidence of the association between schizophrenia and immune functions, elevated levels of inflammation in the dorsolateral prefrontal cortex has been found. To find specific immune patterns in schizophrenia raises the possibility of developing a disease mechanism. Based on the finding of overactive immune system in the brains of schizophrenia, suppression treatment targets in the immune system would cast the future of novel research which introduced a whole new range of treatment possibilities [71].

### BDNF and schizophrenia

**Figure 6:**
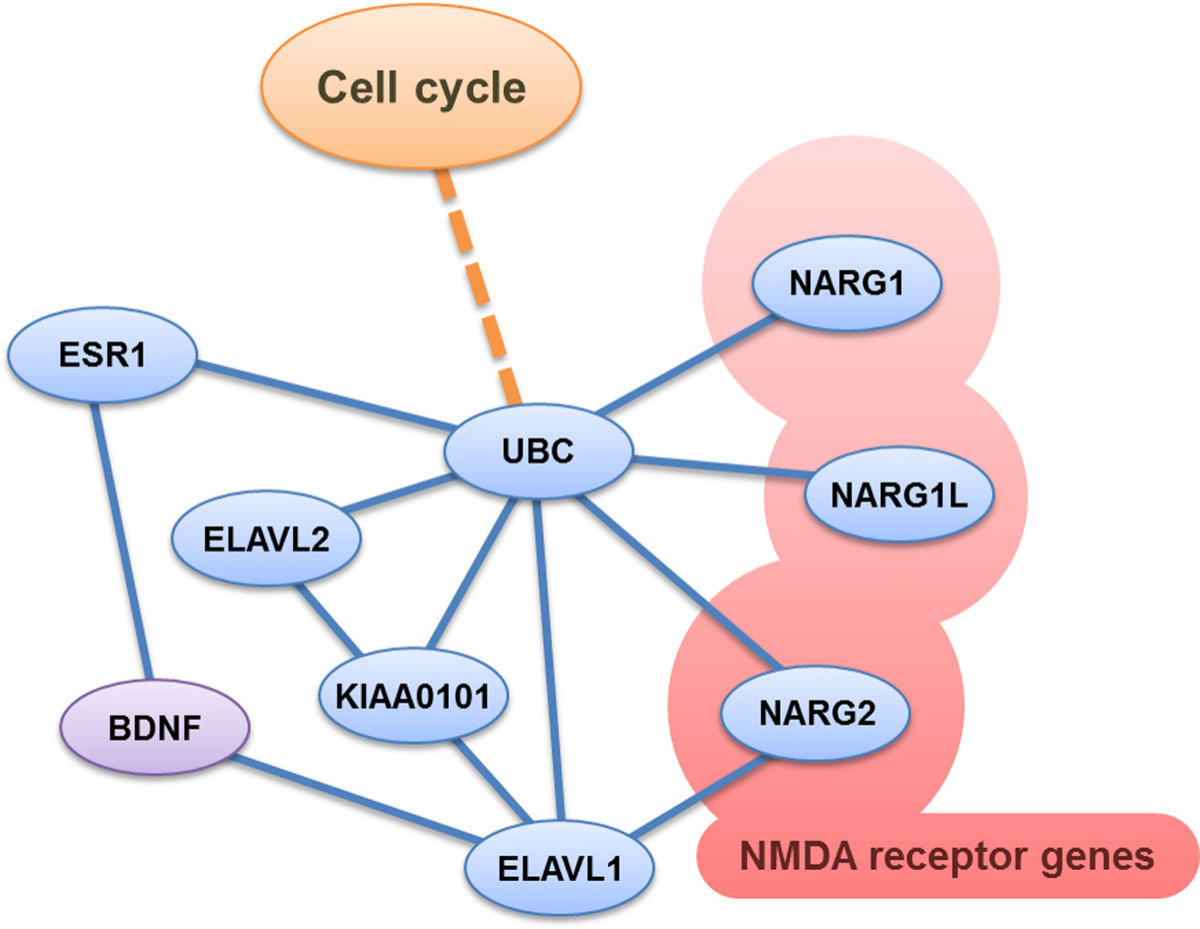
**BDNF-related PPI network**. The PPI of BDNF and NARG2 through EVAVL1 interacts with UBC to cascade the molecular function of cell cycle. Genome-wide association studies indicate significance for the SNP in the ELAVL2 gene associated with schizophrenia. The ELAVL family of RNA-binding proteins regulates gene expression with the implication of biological processes of cancer, and plays an important role in schizophrenia. The KIAA0101 gene interacts with NFkappaB which is important in the BDNF pathway. KIAA0101 is critical in modulating schizophrenic pathway.

Brain-derived neurotropic factor (BDNF) plays an important role in the susceptibility of schizophrenia which is involved in the neurodevelopmental abnormalities of the brain and influences the neuroplasticity in schizophrenia [72-74]. Low BDNF is associated with schizophrenia [75,76]. N-methyl-d-aspartate(NMDA) receptor dysfunction mediated glutamatergic system underlying abnormalities in serum BDNF level and NMDA receptor hypofunction contribute to one of the etiology of schizophrenia [77,78]. Although BDNF does not appear in either the SHCN or the clique network, a simple BDNF network (Figure 6) is proposed and postulated the important mechanism for schizophrenia.

BDNF binds to the TrkB receptor with the presynaptic glutamate to the NMDA receptor activate cascades of PI3K, Akt, and Ras pathway, which formulates the PPI of BDNF and NARG2 through EVAVL1 [78,79] then interacts with UBC to cascade the molecular function of cell cycle. Genome-wide association studies indicate significance for the SNP in the ELAVL2 gene associated with schizophrenia [80]. The extent to which the ELAVL family of RNA-binding proteins regulates gene expression with the implication of biological processes of cancer plays an important role in schizophrenia.

### KIAA0101 and schizophrenia

The KIAA0101 gene has the highest centrality and closeness. KIAA0101 has been observed in a variety of human malignancies and plays a key factor in DNA repair and apoptosis in cell cycle regulation. High-level KIAA0101 expression was also identified as an independent prognostic factor for determining postoperative adjuvant treatments for non-small cell lung carcinoma[81].

The over-expression of KIAA0101 was involved in tumor progression through inhibiting the transcriptional activity of the TP53 gene [82]. KIAA0101 functions as a regulator, promoting cell survival in hepatocellular carcinoma through the regulation of TP53. Suppression of the KIAA0101 function is likely to develop novel cancer therapeutic drugs. In SHCN, KIAA0101 interacts with RUVBL2 which is over-expressed in schizophrenic genes alone with NME2 (yellow region in Figure 2), which indicates the important role in the modulation of disease genes. RUVBL2 is a novel repressor of ARF transcription, ARF is the second most commonly inactivated tumor suppressor gene behind TP53. The genes including KIAA0101, RUVBL2, ARF and TP53 are crucial for schizophrenia.

The KIAA0101 gene is an important cancer gene. In fact, it has PPIs with many other schizophrenic candidate genes. It indirectly interacts with the TP53 gene through the interaction with RUVBL2 and ARF genes [83]. The KIAA0101 gene also interacts with NFkappaB which is important in the BDNF pathway [84]. Through the observation of the PPI network, it is postulated that the KIAA0101 is critical in the modulation of schizophrenic pathways.

## Conclusions

It is not clear that cross-talk among various schizophrenic candidate genes is essential for the explanation of the etiology of schizophrenia. The aim of this research is to evaluate the candidate genes chosen from significant over- and under-expression genes of schizophrenia and hepatocellular carcinoma. The SHCGene formulates the SHCN, including the QQPPI, L1PPI and clique network as a major approach for the discovery of potential complexes and pathways. Investigation of potential schizophrenic pathways with the IL-2/TREM-1 pathway reveals possible complexes or drugs responsible for novel treatment of schizophrenia and hepatocellular carcinoma.

## Competing interests

The authors declare that they have no competing interests.

## Authors' contributions

KCH interpreted the results, drafted the manuscript, and contributed to the design of the bioinformatics analysis tools. SAL programmed the bioinformatics analysis tools and carried out the data analysis. KCY, HL and WKL assisted in the interpretation of results. SAL, TTHT and CYK conceived the study and participate in coordination and management of the research project.

## Supplementary Material

Additional file 1 The SHCGene contains the over- and under- expression candidate genes from the BA22 sample and the Roessler liver 2 sample Click here for file

Additional file 2 **The schizophrenia candidate genes and tumor suppression genes are compared from different literature reviews**. Sun J. et al. (2008), Allen NC. et al. (2008), Ayalew M. et al. (2012), BA22 microarray and hepatocellular carcinoma samples overlap with 716 genes in tumor suppression genes which implicate that schizophrenia candidate genes related to the tumor suppression genes share a common biological regulation. Click here for file

Additional file 3 **The co-expressed mediator genes generated from SHCN L1PPI**. The common mediators of the L1PPI networks of Sun J. et al.(2008), Allen NC. et al. (2008), Ayalew M. et al. (2012) and BA22 microarray samples. The results implicate the importance of mediator genes rather than query genes as key roles of disease susceptibility. Click here for file

Additional file 4 **The pathways of BA22 and Roessler liver 2 samples are ranked by the p-value and FDR-adjusted p-value by the Benjamini-Hochberg procedure**. Click here for file

## Declarations

Publication of this research was funded by Project NSC100-2218-E-424-001 of National Science Council, Taiwan.

This article has been published as part of *BMC Genomics *Volume 14 Supplement 5, 2013: Twelfth International Conference on Bioinformatics (InCoB2013): Computational biology. The full contents of the supplement are available online at http://www.biomedcentral.com/bmcgenomics/supplements/14/S5.
